# Equilibria between the K^+^ binding and cation vacancy conformations of potassium channels

**DOI:** 10.1007/s13238-019-0609-0

**Published:** 2019-02-25

**Authors:** Yao He, Bo Zhang, Hao Dong, Penglin Xu, Xiaoying Cai, Ting Zhou, Mu Yu, Jun Liang, Xiao Zheng, Changlin Tian

**Affiliations:** 1 Hefei National Laboratory for Physical Sciences at the Microscale and School of Life Sciences, University of Science and Technology of China, Hefei, 230027 China; 2 Kuang Yaming Honors School and Institute for Brain Sciences, Nanjing University, Nanjing, 210023 China; 3 Hefei National Laboratory for Physical Sciences at the Microscale and School of Chemistry and Material Sciences, University of Science and Technology of China, Hefei, 230026 China

**Dear Editor,**

Potassium channels are integral membrane proteins that selectively conduct K^+^ ions across cell membrane (Hille, 2001). They play essential roles in maintaining cellular ionic homoeostasis and generating action membrane potentials in excitable cells. The mechanism of K^+^ selectivity has been evaluated in many biophysical and physiological studies (Zhou et al., 2001; Liu et al., 2015; Schewe et al., 2016). A highly conserved signature sequence, TVGYG, in the selectivity filter of potassium channels (e.g., KcsA) is known to coordinate K^+^ ions (Zhou et al., 2001). Carbonyls of these residues point toward the pore, forming four continuous ion binding sites (S1–S4) and resulting in higher selectivity for K^+^ over Na^+^ (Zhou et al., 2001). The NaK channel from *Bacillus cereus* is a non-selective cation channel that shares high structural homology with KcsA (Shi et al., 2006). Owing to a distinctive primary sequence of _63_TVGDG_67_, the selectivity filter of NaK preserves only two ion binding sites, allocated similarly as S3 and S4 in KcsA (Alam and Jiang, 2009a, b). Remarkably, the D66Y and N68D double mutations of NaK channel (Fig. S1) transform it into a K^+^ selective channel (termed as NaK2K) (Sauer et al., 2013). Crystal structure of NaK and NaK2K have revealed distinct binding coordination of Na^+^ and K^+^ ions in their selectivity filter (Figs. S2 and S3) (Alam and Jiang, 2009a, b; Sauer et al., 2013). However, dynamics of the NaK and NaK2K selectivity filter with the binding of Na^+^ or K^+^ are still elusive. Especially, it has been known that the membrane environment is highly diverse from detergent micelles, which was considered to influence the structure and function of membrane proteins dramatically (Cross et al., 2011). Thus, it is necessary to study the cation and binding properties of NaK NaK2K channels in lipid bilayers.

Solid-state NMR (ssNMR) is frequently applied to study the structure and dynamics of membrane proteins in lipid bilayers (Lange et al., 2006; Hu et al., 2010). Chemical shift and line shape of ssNMR resonance are sensitive to local variations surrounding the detection nuclei. Therefore, the ^17^O or ^13^C isotope labelled carbonyl groups in the selectivity filter of NaK and NaK2K can act as perfect NMR probes to reflect subtle environmental variations, including nearby ions binding as well as the dynamics of the selectivity filter themselves. Nevertheless, it is rather difficult to acquire ^17^O NMR signals, simply because of the difficulties in site-specific isotope labelling and the complicated date interpretation of quadruple ^17^O spins (Wu 2016). In this work, site-specific Val64-^13^C(O) resonances of NaK and NaK2K at different cation conditions were acquired to provide insight into the ion binding process for both non-selective NaK channel and K^+^ selective NaK2K channel.

Considering there is only a single pair of Valine-Glycine dipeptide pair along the primary sequence of NaK or NaK2K (Fig. 1A), the site-specific Val64-^13^C(O) signals could be obtained through combination of two amino acids (^13^C(O)-Val and ^15^N-Gly) specific labelling in NaK or NaK2K samples, and using NCO double cross polarization (DCP) ssNMR pulse sequences (Fig. 1C). The amino acids labelled NaK and NaK2K were expressed in M9 medium, purified in the n-decyl-β-D-maltoside (DM), and reconstituted into DMPC/DMPG (3:1) lipid bilayers. The tetrameric NaK and NaK2K channel were still maintained in proteoliposomes, as shown by SDS-PAGE (Fig. S4B). Single-channel recordings verified the well-folded and functional NaK channel in lipids (Fig. S4C). The ssNMR experiments were performed on a 600 MHz wide-bore spectrometer. Using the NCO DCP pulse sequence (Fig. 1C), magnetization of the ^15^N-Gly was initially built up through cross polarization transfer from ambient protons, then transferred to a directly bonded ^13^C(O) spin, thus resulting in high specificity for the dipeptide ^13^C(O)-Val64-^15^N-Gly65 (Fig. 1B). The NCO DCP also effectively suppressed the natural abundance ^13^C signals, especially from lipids (Figs. 1D, S6 and S7).

**Figure 1:**
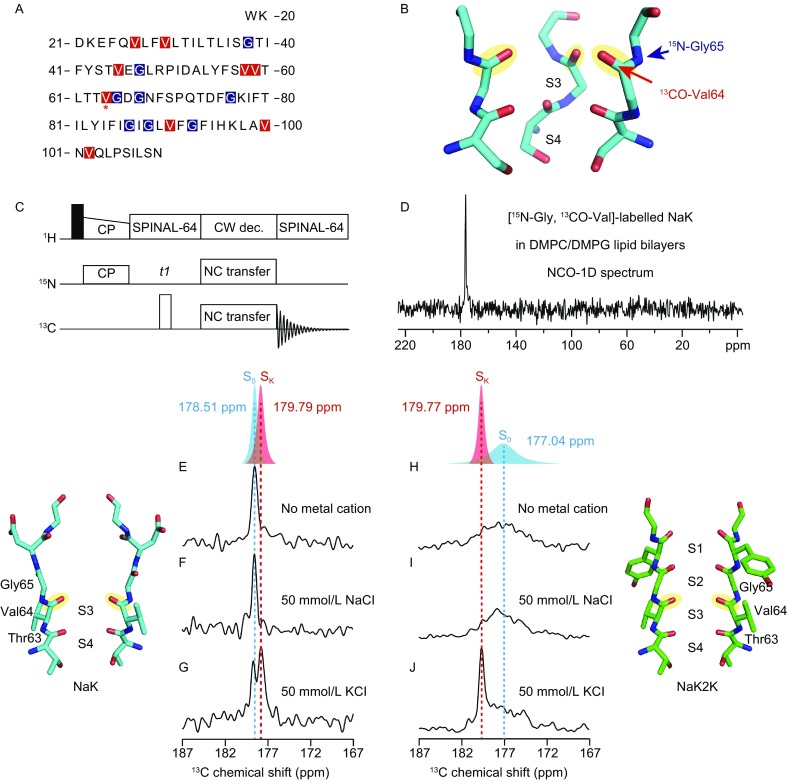
Site-specific isotope labelling strategy and ssNMR analysis of 13C(O)-Val64 resonances at different cation conditions. (A) Amino acid sequence of the NaK channel. Val64 was marked with asterisk. (B) The carbonyl groups of Val64 were shaded in yellow. The front subunit was removed for clarity. (C) Pulse sequence of NCO DCP experiments. Open and filled bars represent 180° and 90° pulse, respectively. (D) One-dimensional ^13^C-NCO spectrum of [^15^N-Gly, ^13^CO-Val]-labelled NaK in DMPC/DMPG lipid bilayers. [^15^N-Gly, ^13^CO-Val]-labelled NaK in buffer without any metal cation (E), in presence of 50 mmol/L NaCl (F) or 50 mmol/L KCl (G). [^15^N-Gly, ^13^CO-Val]-labelled NaK2K in buffer without any metal cation (H), in presence of 50 mmol/L NaCl (I) or 50 mmol/L KCl (J). The carbonyl groups of Val64 were shaded in yellow on the structure of NaK and NaK2K selectivity filter

First, we collected one-dimensional NCO spectra of [^15^N-Gly, ^13^CO-Val]-labelled NaK or NaK2K in lipid bilayers without any metal cation and obtained S_0_ resonances for NaK and NaK2K, respectively (Fig. 1E and 1H). Excluding the binding of Na^+^ or K^+^ in both samples, the S_0_ resonances of NaK and NaK2K could represent specific cation vacancy conformation of NaK and NaK2K (i.e., without any metal cation coordinated in the selective filter), respectively. The single sharp S_0_ resonance of NaK was observed at 178.51 ppm (Fig. 1E) and line width of this peak was only 0.6 ppm (full-width at half-height, FWHH), indicating the vacancy conformation of NaK was rigid and stable. However, the S_0_ resonance of NaK2K was quite broad (Fig. 1H), indicating a non-homogenous conformation without presence of either Na^+^ or K^+^.

To further characterize the difference binding properties of Na^+^ and K^+^ in the selectivity filter of NaK or NaK2K, NCO spectra of [^15^N-Gly, ^13^CO-Val]-labelled NaK or NaK2K in the presence of 50 mmol/L NaCl or 50 mmol/L KCl were acquired. As shown in Fig. 1F and 1I, the Val64-^13^C(O) resonances of NaK or NaK2K in the presence of 50 mmol/L NaCl was quite similar as the S_0_ resonance of NaK or NaK2K (Fig. 1E and 1H), respectively. Since it is known that the ^13^C resonances are sensitive to local environment (Hu et al., 2010), the almost unchanged site-specific Val64-^13^C(O) spectra indicated that there must be no Na^+^ binding around the Val64 carbonyl groups of either NaK or NaK2K. This was contradictory to the observed electron density at S3 site in the crystal structure of NaK_Na^+^ complex (PDB number 3e89) (Alam and Jiang, 2009a, b). Nevertheless, authors of the crystallography studies have mentioned that it was difficult to distinguish the electron densities between Na^+^ and water molecules (Sauer et al., 2013). The observed electron density at S3 site in the crystallography studies might be generated by a water molecule, instead of a binding Na^+^ ion, or a non-specific Na^+^ ion trapped in the low temperature (~100 K) condition during X-ray diffraction (Fowler et al., 2008).

In the NCO spectra of [^15^N-Gly, ^13^CO-Val]-labelled NaK or NaK2K in the presence of 50 mmol/L KCl (Fig. 1G and 1J), two resonances were observed for each sample. One resonance was similar as S_0_, while the other resonance could be assigned as S_k_—the Val64-^13^C(O) resonance of NaK or NaK2K under the influence of K^+^. Chemical shifts of the well-resolved S_K_ resonances were 177.79 ppm and 179.77 ppm for NaK and NaK2K respectively (Fig. 1G and 1F). The presence of S_k_ strongly indicated the binding of K^+^ in the selectivity filter of NaK and NaK2K. Meanwhile, observation of both S_0_ and S_K_ implied that the cation vacancy conformation (S_0_ resonance) and K^+^ binding conformation (S_K_ resonance) existed simultaneously in the selectivity filter of NaK or NaK2K channels. Furthermore, similar integrated area of the S_0_ and S_k_ resonances indicated almost equal proportions of the two conformations. These results might suggest the K^+^ flux coming through (S_k_) or away from (S_0_) the Val64-C(O) site, which was consistent with the proposed K^+^ selectivity and flux model in KcsA (Morais-Cabral et al., 2001).

As shown in Fig. 1H and 1J, the line shape of S_K_ and S_0_ resonances were quite different for NaK2K, suggesting pronounced dynamics variations of the NaK2K Val64 backbone carbonyl during K^+^ binding process. May due to the structure difference in the hydrogen bond network between pore helix and selectivity filter, NaK2K vacancy conformation shows a slower dynamic in the filter than the KcsA C-type inactivation conformation, which shows a set of sharp peaks on solid state NMR (Bhate et al., 2010). To validate this possibility, molecular dynamics (MD) simulations were conducted with the duration of ~100 ns for both NaK2K_Tris (as S_0_ resonance) and NaK2K_K^+^ (as S_k_ resonance) systems (Fig. 2A). Swing motion of the Val64 carbonyl groups was characterized with the θ_Val64-C(O)_ angle, which was formed by the two C=O vectors from MD simulation and the reference crystal structure (Fig. 2B). The optimal θ_Val64-C(O)_ angle in the NaK2K_K^+^ system was 5°, while that angle in the NaK2K_Tris system was 24° (Fig. 2C), indicating different Val64-C(O) orientation with respect to the normal direction of the channel pore. Meanwhile, the angle distribution of θ_Val64-C(O)_ in the NaK2K_K^+^ system (0° to 20°) were much narrower than that of NaK2K_Tris system (5° to 50°), demonstrating the more stable and rigid conformation of Val64-C(O) upon the binding of K^+^ (Fig. 2C). As references, the angle distribution of θ_Thr63-C(O)_ in both systems were also analyzed and only minor variations were observed (Fig. 2D). The correlation analysis of θ_Val64-C(O)_ and θ_Thr63-C(O)_ angels in either NaK2K_K^+^ (Fig. S8A) or NaK2K_Tris (Fig. S8b) system also suggested the high sensitivity of Val64-C(O) than Thr63-C(O) during K^+^ binding in the selectivity filter of NaK2K. Overall, our ssNMR line shape analysis and parallel MD simulation data highly verified the previous hypothesis that the binding of K^+^ ions could stabilize the selectivity filter of NaK2K, which might result in the “conducting” state.

**Figure 2:**
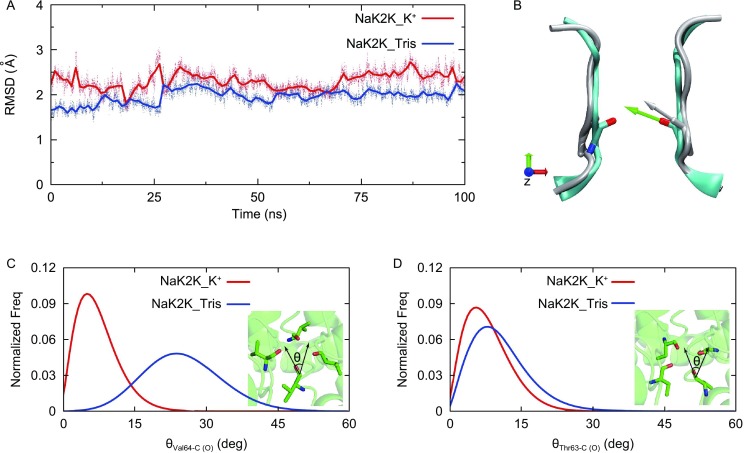
Dynamics of the NaK2K Val64 carbonyl groups during K^+^ binding process. (A) RMSD of the NaK2K_K^+^ and NaK2K_Tris systems during MD simulation. (B) θ_Val64-C(O)_ angle was formed by the two C=O vectors from MD simulations (grey) and the reference crystal structure (cyan). (C) Distribution of θ_Val64-C(O)_ in NaK2K_K^+^ system (red) and in NaK2K_Tris system (blue). (D) Distribution of θ_Thr63-C(O)_ in NaK2K_K^+^ system (red) and in NaK2K_Tris system (blue)

In summary, we have obtained the site-specific Val64-^13^C(O) spectra of NaK and NaK2K in lipid bilayers through the combination of double amino acid specific labeling and NCO DCP ssNMR experiments. The analysis of Val64-^13^C(O) resonances at different Na^+^/K^+^ conditions indicated no Na^+^ ion binding in the vicinity of NaK-Val64-C(O) and suggested that both K^+^-selective NaK2K channel and non-selective NaK channel preferred to bind K^+^ ions in the central part of the selectivity filter. We also observed the distinctive equilibria between the K^+^ binding conformation and cation vacancy conformation in selectivity filter of NaK or NaK2K through ssNMR methods. The experimental line-width analysis and MD simulations verified the conformational stabilization of NaK2K selective filters upon the binding of K^+^ ions. The two conformations might show in ion channels simultaneously. The S_k_ conformation provide the K^+^ conduction pathway which dehydrated K^+^ can pass through in single file. The S_0_ conformation may conduct partially hydrated Na^+^ (Kuhlbrandt, 2016), which leads a similar chemical environment for V64 in the vacancy conformation with water molecules. Methods in this study could be applied to shed more insights into the ion binding process of ion channels in native-like membrane environments and to illustrate physical chemistry mechanisms of other tetrameric ion channels.

## Electronic supplementary material

Below is the link to the electronic supplementary material.

Supplementary material 1 (PDF 1394 kb)
