# Supplementary material for: Equilibria between the K+ binding and cation vacancy conformations of potassium channels
Source: Protein Cell. 2019 Feb 25;10(7):533–7. doi: 10.1007/s13238-019-0609-0 (PMC6588643; doi:10.1007/s13238-019-0609-0)
Supplement: Supplementary file 1 — Supplementary material 1 (PDF 1394 kb) [file 13238_2019_609_MOESM1_ESM.pdf]

Equilibria between the K^+ binding and cation vacancy conformations of potassium channels

Yao He[1,‡], Bo Zhang[1,‡], Hao Dong,[2,*] Penglin Xu[3], Xiaoying Cai[1], Ting Zhou[3], Mu Yu[1], Jun Liang[1], Xiao Zheng,[3,*] and Changlin Tian[1,*]

Email: donghao@nju.edu.cn; xz58@ustc.edu.cn; cltian@ustc.edu.cn

1. Hefei National Laboratory for Physical Sciences at the Microscale and School of Life Sciences, University of Science and Technology of China, Hefei 230027, China
2. Kuang Yaming Honors School and Institute for Brain Sciences, Nanjing University, Nanjing, 210023, China
3. Hefei National Laboratory for Physical Sciences at the Microscale and School of Chemistry and Material Sciences, University of Science and Technology of China, Hefei 230026, China

‡ These authors contributed equally to this work.

Experiment Section

Protein Expression and Purification. The NaK and NaK2K genes with their N-terminal 19 residues removed were cloned into a modified pET28a vector (Novagen).[1] The proteins were expressed in *E. coli* BL21(DE3)-Gold cultures. Cells were grown in M9 minimal medium supplemented with specific isotope labelled amino

acids at 37 °C until the OD600 value reached 0.6—0.8, then induced using 0.2 mM isopropyl b-D-thiogalactopyranoside (IPTG, Sigma) at 25 °C for an additional 12 h. The cells were harvested by centrifugation, resuspended in lysis buffer (70 mM Tris-HCl, pH 8.0, 300 mM NaCl) and lysed by sonication. Cell debris was removed by centrifugation and the supernatant was solubilized in 1% (w/v) n-decyl-β-D-maltoside (DM, Anatrace) at 4 °C for 3 h. The over-expressed proteins were purified using Ni^{2+}-NTA affinity column (QIAGEN) and eluted by a buffer of 20 mM Tris-HCl, pH 8.0, 200 mM NaCl supplemented with 0.2% (w/v) DM and 300 mM imidazole. The proteins were further purified on a Superdex 200 (10/300) gel filtration column (GE healthcare) in a buffer of 20 mM Tris-HCl, pH 8.0, 200 mM NaCl, 0.2% (w/v) DM. The fractionally collected proteins were concentrated using an Amicon Ultra filter (10,000 MWCO, Millipore) and analyzed by SDS-PAGE.

Circular Dichroism (CD) Spectroscopy. The purified NaK proteins in DM micelles were diluted to 0.10 mM with PBS buffer (50 mM NaH_2PO_4/Na_2HPO_4, pH 8.0). CD measurements were performed on a Jasco-810 spectropolarimeter at 298 K. Spectra were recorded over a wavelength range of 190–280 nm using a cuvette of 1.0 mm path length at a scanning speed of 20 nm/min and subjected to 10 scans. Acquired data were normalized by subtracting the baseline recorded for the buffer only.

Reconstitution of Proteoliposome. Powders of 1,2-dimyristoyl-*sn*-glycero-3-phosphocholine (DMPC) and

1,2-dimyristoyl-*sn*-glycero-3-phospho-[1'-rac-glycero] (DMPG, sodium salt) lipids (Avanti Polar Lipids, AL, US) were mixed in chloroform with molar ratio of 3:1 (DMPC:DMPG). The solution was gently dried under nitrogen flow and then placed under a high vacuum overnight to further evaporate any residual solvent. The lipid film was rehydrated with binding buffer (20 mM Tris, pH 8.0, 200 mM NaCl) to yield a final concentration of approximately 10 mg/ml, then dispersed by vigorous stirring followed by at least 10 freeze-thaw-sonication cycles until the solution was clear. The resulting solution was extruded 20 times through a 100 nm polycarbonate membrane filter (Whatman, Newton, MA) mounted on a mini-extruder.

The purified proteins and the extruded lipids were mixed together and dialysis against 500-fold excessive buffer for a total of 96 hours and the buffer was changed every 12 hours. The protein:lipid ration was 1:50 (mol/mol) for ssNMR experiments and 1/1000 (mol/mol) for single-channel conductance measurements. Three different types of buffer were used in the reconstitution of NaK/NaK2K proteoliposomes at different ion conditions: 20 mM Tris-HCl, pH 8.0, 50 mM NaCl for the samples in the presence of 50 mM Na^+; 20 mM Tris-HCl, pH 8.0, 50 mM KCl for the samples in the presence of 50 mM K^+; 20 mM Tris-HCl, pH 8.0 for the samples in absence of any mental cation. All chemicals used in the experiments were purchased from Sigma-Aldrich.

Single-Channel Conductance Measurement. After dialysis, the protein/lipid mixture was extruded 20 times through a 100 nm polycarbonate membrane filter (Whatman,

Newton, MA) by the Avanti Mini-Extruder to make unilamellar liposome vesicles. Channel conductance measurements in planar lipid bilayer were conducted using Ionovation Compact (Osnabrück, Germany). Two polycarbonate compartments in volume of 1.2 mL were separated by a TEFLON foil with 25 μm thickness and 50−100 μm aperture diameter. The artificial lipid bilayer was formed onto the aperture through method of painting. The cis-chamber was filled with 15 mM NaCl, 150 mM KCl, 5 mM Mops, 5 mM Tris-HCl, pH 7.0, and the trans-chamber with 150 mM NaCl, 15 mM KCl, 5 mM Mops, 5 mM Tris-HCl, pH 7.0. Voltage was applied across the bilayer using Ag/AgCl electrodes immersed in each chamber. With the grounded cis-compartment, a positive potential indicating a higher potential in the trans-chamber was applied. Planar lipid bilayer formation was monitored optically or by capacitance measurements. After successful formation of a stable bilayer in the aperture, NaK proteoliposomes were added to the trans-chamber next to the bilayer. Fusion of protein and planar lipid bilayer was detected through observation of channel conductance. Signal acquisition and analysis were performed using the pCLAMP software (Axon Instruments). Single-channel conductance events were identified automatically and analyzed using Clampfit 10.0 software (Axon Instruments).

Solid-State NMR (ssNMR) Experiments. After dialysis, the proteoliposome samples were collected by ultracentrifugation at 300,000 ×g and 4 °C for 2 hours. The pellets were then loaded into a 3.2 mm magic-angle spinning (MAS) rotors and subjected to ssNMR researches. All ssNMR spectra were recorded on a Bruker Ascend 600 MHz

(14.1 T) wide-bore spectrometer equipped with a 3.2 mm E-free triple-resonance (^{1}H, ^{13}C, ^{15}N) MAS probe. The spinning frequency during experiments was 12 kHz. The probe temperature was kept at 270 K and the sample temperature was around 10 °C. Chemical shift of all spectra was calibrated using DSS (sodium salt of 2,2-dimethyl-2-sila- pentane-5-sulphonic acid) as an external reference.[2] Typical π/2 pulses were 3 μs for ^{1}H, 5 μs for ^{13}C, and 6 μs for ^{15}N, respectively. In NCO experiments, the polarization transfers between ^{15}N and ^{13}C was set up through SPECIFIC-CP[3]. High power proton-decoupling using SPINAL64[4] with ^{1}H radio-frequency (RF) of 70 kHz was applied during acquisition and indirect evolution periods. 40,960 scans were recorded with recycle delay of 2.0 s, which resulted in a total acquisition time of 23 h for each NCO spectrum. All spectra were processed and analyzed with TOPSPIN 3.1.

Molecular Dynamics (MD) Calculations. The 1.55 Å resolution X-ray structure of NaK2K (PDB number 3OUF)[5] was used as the starting structure for NaK2K_K^+ and NaK2K_Tris systems. The protein was embedded in a lipid bilayer consisting of randomly placed mixture of 121 DMPC molecules and 40 DMPG molecules, giving DMPC/DMPG molar ratio of 3:1. For the NaK2K_K^+ system, the K^+ ions in the selectivity filter of the crystal structure were kept, with additional K^+ and Cl^- added to neutralize the system. For the NaK2K_Tris system, the K^+ ions in the selectivity filter were removed, and those K^+ in bulk water were replaced with Tris. The initial water molecules, ions and the lipid bilayers were built with CHARMM-GUI[6]. The total

number of atoms in each system was ~ 55000. The CHARMM36 force field was used for protein and lipids.[7] Parameters for Tris were generated by using CGenFF.[8] The TIP3P model was used for water[9]. Periodic boundary conditions were applied, with the particle mesh Ewald method used for treating long-range electrostatic interactions.[10] The van der Waals interactions were smoothly switched off in the range of 10 Å and 12 Å by a switching function. The SHAKE algorithm was applied to constrain all bonds involving hydrogen atoms, which allowed for an integration time step of 2 fs. After equilibration, a 100 ns production run was performed for each system. All calculations were performed in an NPT (constant particles, pressure, and temperature) ensemble at 303.15 K using NAMD 2.9.[11]

Supplementary Figures

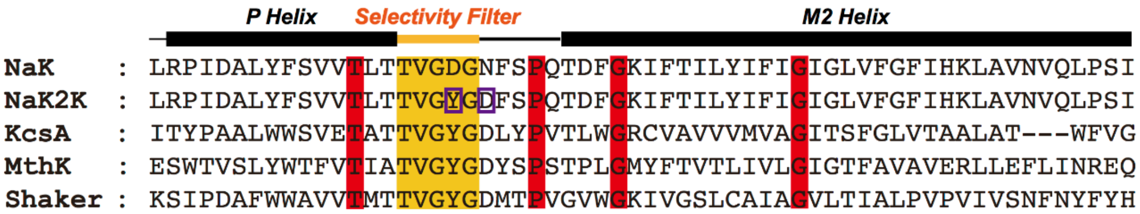

Figure S1. Sequence alignment of NaK, NaK2K and some other potassium channels like KcsA, MthK and Shaker. Conserved residues were shaded in red and selectivity filter residues were shaded in yellow. The mutant residues in NaK2K were marked with purple boxes. Secondary structure assignment was based on the NaKNΔ19 structure (PDB number 3E86).

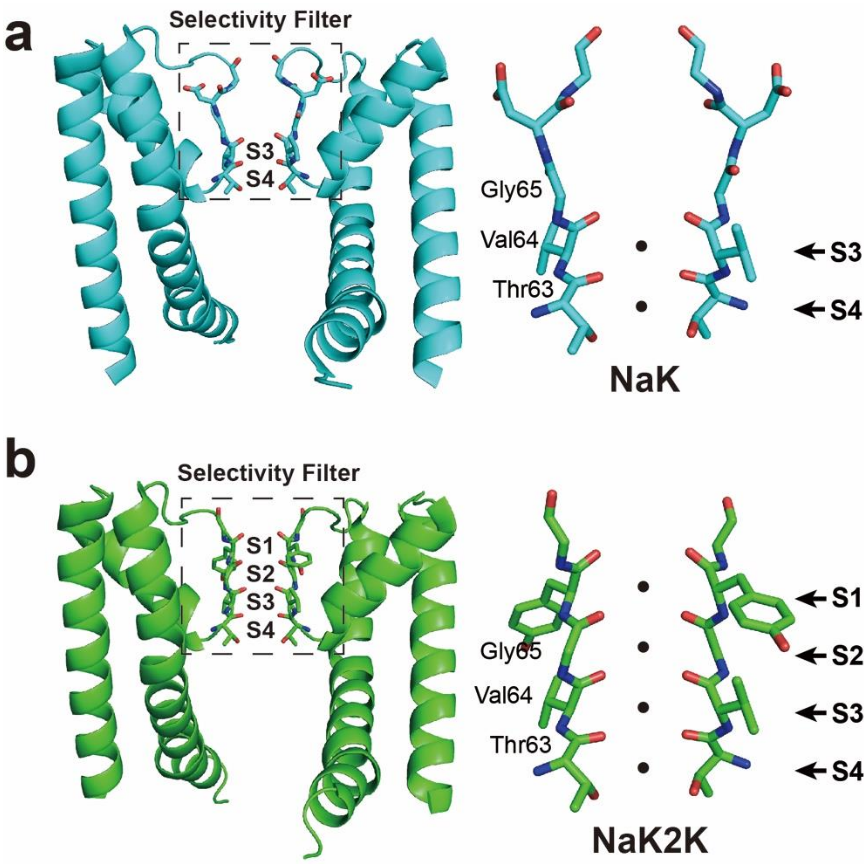

Figure S2. Crystal structure of NaK (cyan, PDB number 3E86) and NaK2K (green, PDB number 3ouf) viewed from the mem-brane with the front and rear subunits removed for clarity. The ion binding sites were labelled as S1-S4 respectively.

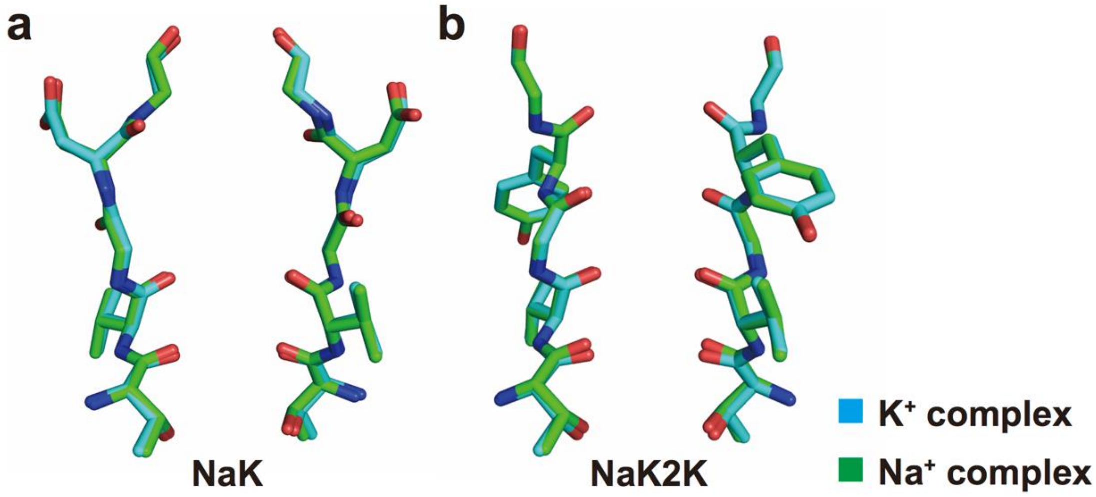

Figure S3. Apart from the different coordinated ions, the selectivity filter structures of NaK (or NaK2K) in conditions with various cations are quite similar. (a) Superimposition of the selectivity filter structures from NaK_Na^+ and NaK_K^+ complexes. RMSD of backbone atoms were 0.178 Å. (b) Superimposition of the selectivity filter structures from NaK2K_Na^+ and NaK2K_K^+ complexes. RMSD of backbone atoms were 0.106 Å.

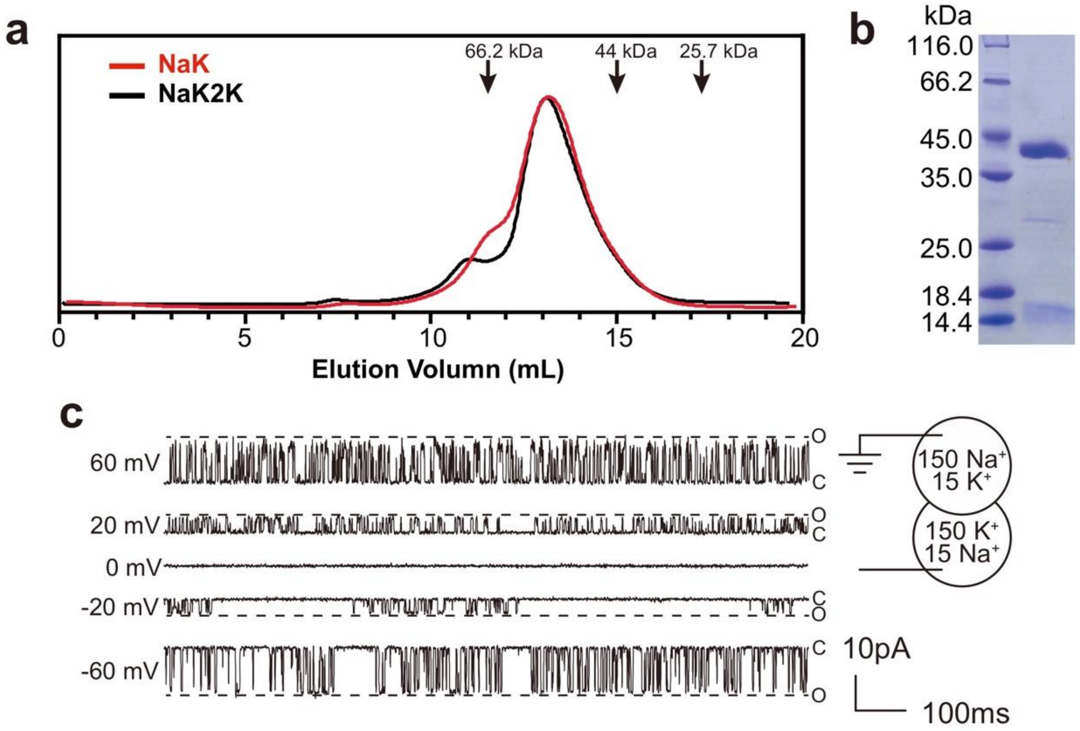

Figure S4. (a) Gel filtration revealed the formation of NaK and NaK2K tetramer in DM micelles. (b) Coomassie-stained SDS-PAGE gel of NaK reconstituted into the DMPC/DMPG lipid bilayers. The band at approximately 37 kDa was the homo-tetramer of NaK. (c) Single-channel conductance of NaK after reconstitution in lipid bilayers at various membrane potentials. Currents were recorded with 15 mM NaCl, 150 mM KCl, 5 mM Mops, 5 mM Tris-HCl, pH 7.0 in the cis-chamber and 150 mM NaCl, 15 mM KCl, 5 mM Mops, 5 mM Tris-HCl, pH 7.0 in the trans-chamber. Solid line: closure (C); dashed line: opening (O).

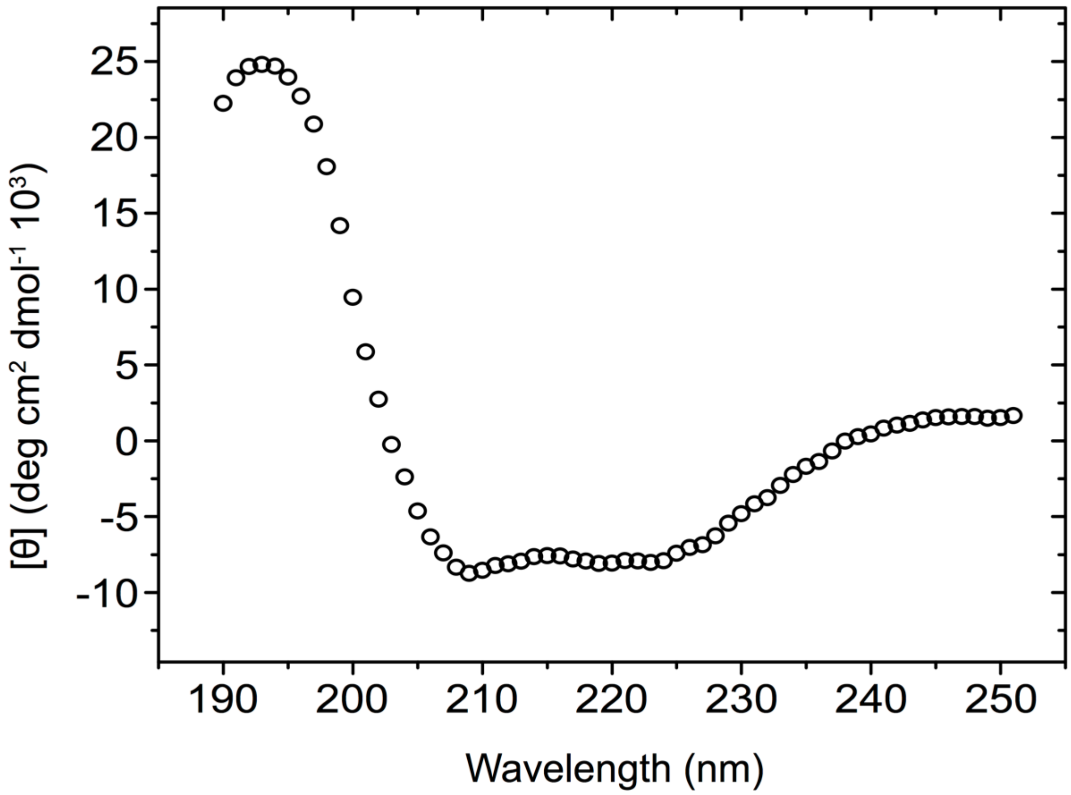

Figure S5. Circular dichroism (CD) spectrum of NaK in DM micelles exhibited the minimum absorption at 208 and 222 nm, indicating the formation of well-defined α-helical structure

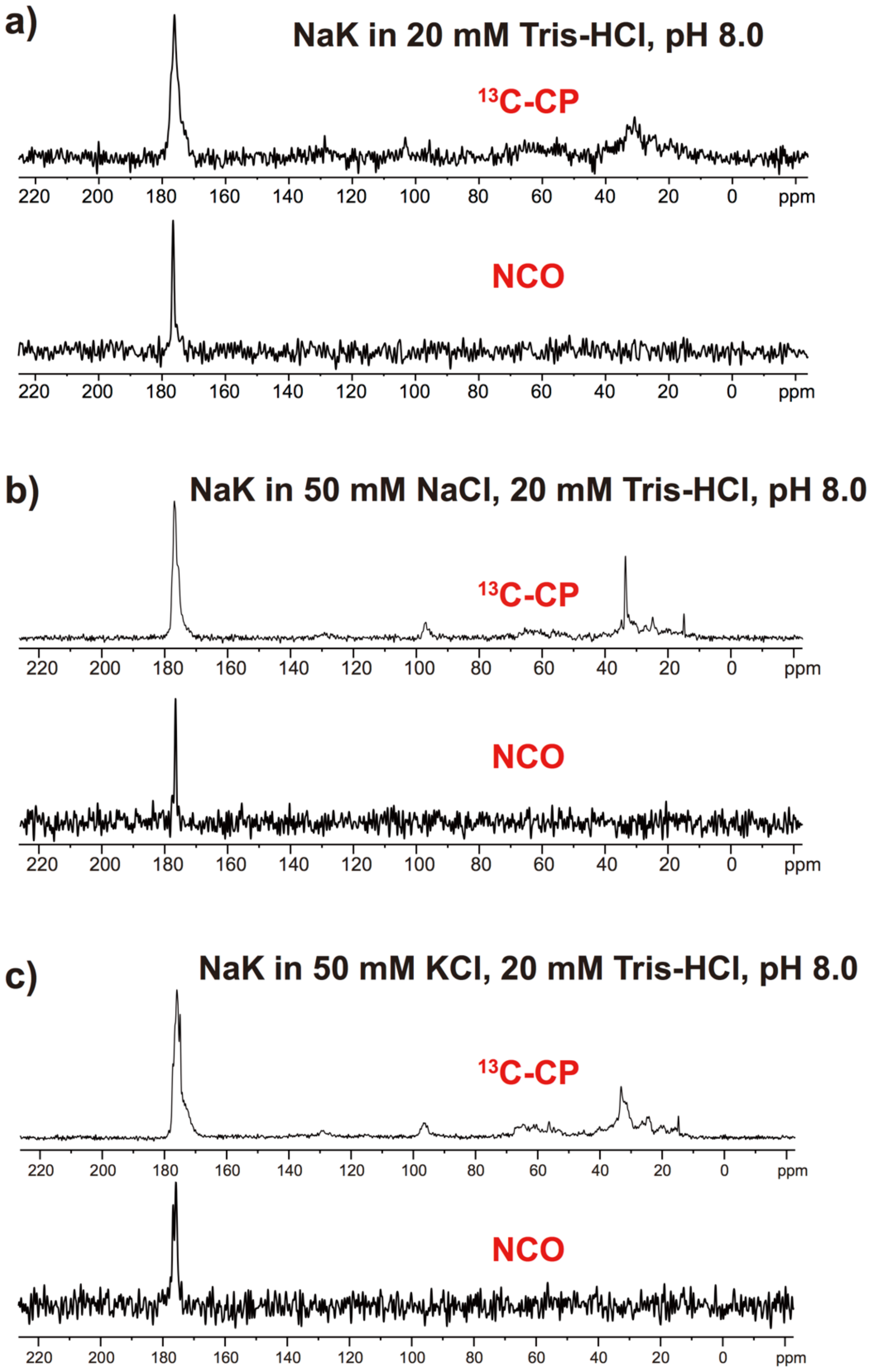

Figure S6. ^{13}C-CP and NCO spectra of the [^{15}N-Gly, ^{13}CO-Val]-labelled NaK proteoliposome in three different buffers.

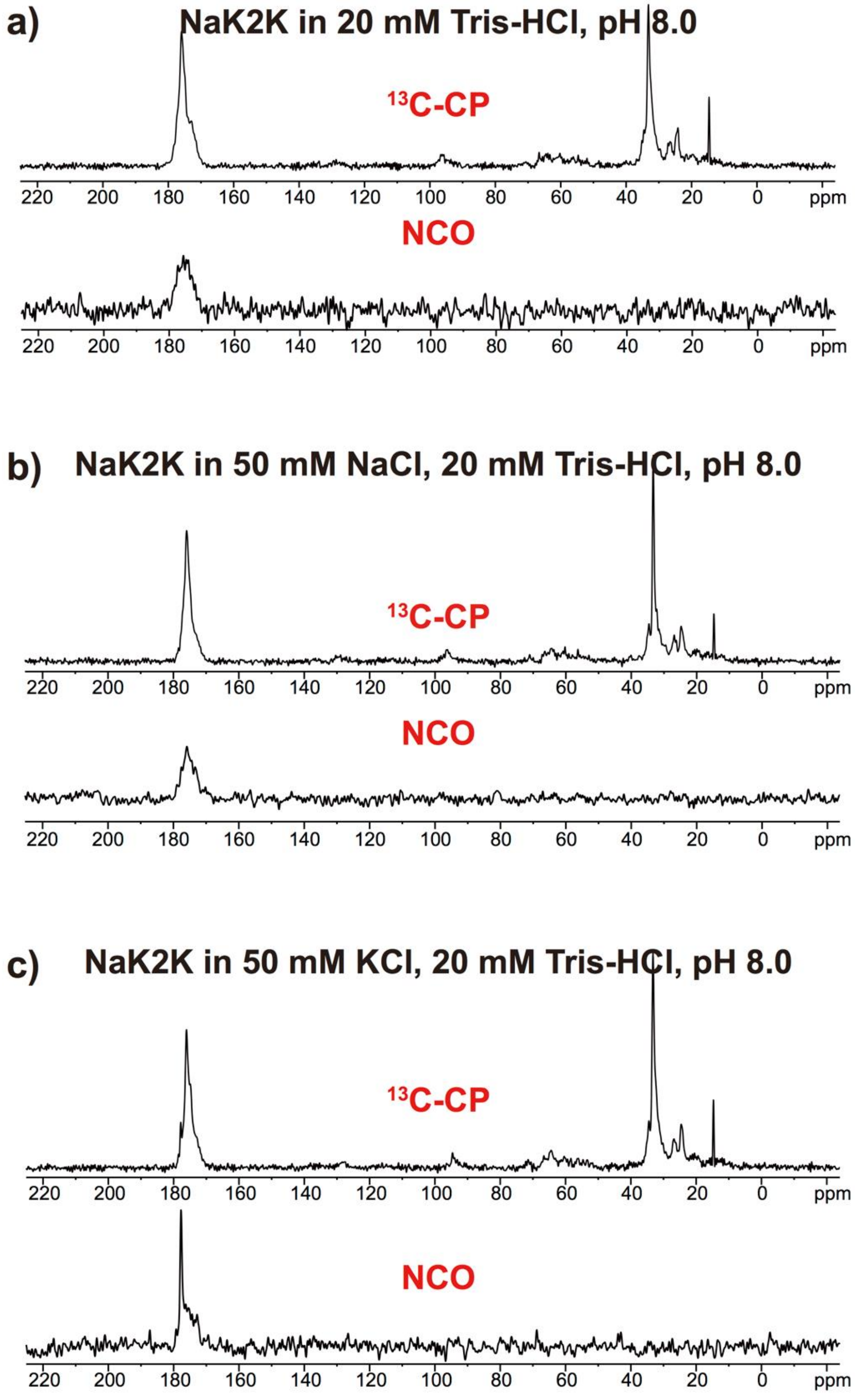

Figure S7. ^{13}C-CP and NCO spectra of the [^{15}N-Gly, ^{13}CO-Val]-labelled NaK2K proteoliposome in three different buffers.

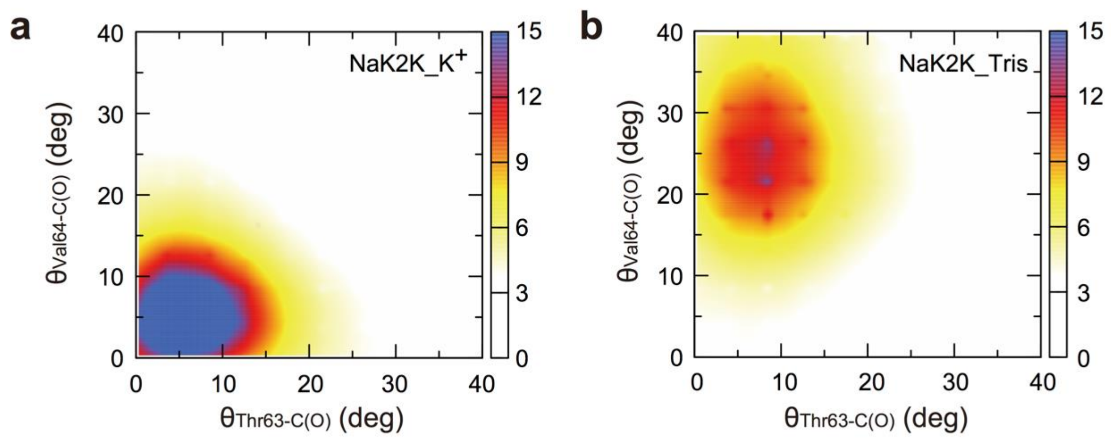

Figure S8. (a) Correlation distribution between $\theta_{Thr63\text{-}C(O)}$ and $\theta_{Val64\text{-}C(O)}$ angles in the NaK2K_K$^+$ system. (b) Correlation distribution between $\theta_{Thr63\text{-}C(O)}$ and $\theta_{Val64\text{-}C(O)}$ angles in the NaK2K_Tris system.

References

[1] S. Liu, P. Lv, D. Li, X. Guo, B. Zhang, M. Yu, D. Li, Y. Xiong, L. Zhang, C. Tian, *Chem. Commun. (Camb.)* **2015**, *51*, 15971-15974.

[2] C. R. Morcombe, K. W. Zilm, *J. Magn. Reson.* **2003**, *162*, 479-486.

[3] M. Baldus, A. T. Petkova, J. Herzfeld, R. G. Griffin, *Mol. Phys.* **1998**, *95*, 1197-1207.

[4] B. M. Fung, A. K. Khitrin, K. Ermolaev, *J. Magn. Reson.* **2000**, *142*, 97-101.

[5] M. G. Derebe, D. B. Sauer, W. Zeng, A. Alam, N. Shi, Y. Jiang, *Proc. Natl. Acad. Sci. USA* **2011**, *108*, 598-602.

[6] J. Lee, X. Cheng, J. M. Swails, M. S. Yeom, P. K. Eastman, J. A. Lemkul, *et al. Journal of chemical theory and computation* **2016**, *12*, 405-413.

[7] a) A. D. MacKerell, D. Bashford, M. Bellott, R. L. Dunbrack, J. D. Evanseck, M. J. Field, *et al. J. Phys. Chem. B* **1998**, *102*, 3586-3616; b) B. R. Brooks, C. L. Brooks, A. D. Mackerell, L. Nilsson, R. J. Petrella, B. Roux, *et al. J. Comp. Chem.* **2009**, *30*, 1545-1614; c) J. B. Klauda, R. M. Venable, J. A. Freites, J. W. O'Connor, D. J. Tobias, C. Mondragon-Ramirez, *et al. J. Phys. Chem. B* **2010**, *114*, 7830-7843.

[8] a) K. Vanommeslaeghe, E. Hatcher, C. Acharya, S. Kundu, S. Zhong, J. Shim, *et al. J. Comp. Chem.* **2010**, *31*, 671-690; b) W. Yu, X. He, K. Vanommeslaeghe, A. D. MacKerell, *J. Comp. Chem.* **2012**, *33*, 2451-2468.

[9] W. L. Jorgensen, J. Chandrasekhar, J. D. Madura, R. W. Impey, M. L. Klein, *J. Chem. Phys.* **1983**, *79*, 926-935.

[10] U. Essmann, L. Perera, M. L. Berkowitz, T. Darden, H. Lee, L. G. Pedersen, *J. Chem. Phys.* **1995**, *103*, 8577–859.

[11] J. C. Phillips, R. Braun, W. Wang, J. Gumbart, E. Tajkhorshid, E. Villa, *et al. J. Comp. Chem.***2005**, *26*, 1781-1802.
